# Production of cyathane type secondary metabolites by submerged cultures of *Hericium erinaceus* and evaluation of their antibacterial activity by direct bioautography

**DOI:** 10.1186/s40694-015-0018-y

**Published:** 2015-12-22

**Authors:** T. Shen, G. Morlock, H. Zorn

**Affiliations:** 1 Institute of Food Chemistry and Food Biotechnology, Justus Liebig University Giessen, Heinrich-Buff-Ring 17, 35392 Giessen, Germany; 2 Institute of Nutritional Science, and Interdisciplinary Research Center for Biosystems, Land Use and Nutrition, Justus Liebig University Giessen, Heinrich-Buff-Ring 26-32, 35392 Giessen, Germany

**Keywords:** *Hericium erinaceus*, Cyathane type diterpenoids, Erinacine C, Erinacine P, HPTLC, TLC-MS interface, *Aliivibrio fischeri*

## Abstract

**Background:**

Fungi of the phylum Basidiomycota are well-known to form a broad spectrum of biologically active secondary metabolites, especially low molecular weight compounds such as terpenoids. *Hericium erinaceus* produces various cyathane type diterpenoids including erinacines. However, no quantitative data and production kinetics have been reported on the biosynthesis of the erinacines C and P in submerged cultures. In the present study, the production of erinacine C was optimized, and the product formation kinetics as well as the antimicrobial activity were studied by high-performance liquid chromatography (HPLC), high-performance thin-layer chromatography (HPTLC) and direct bioautography.

**Results:**

Oatmeal and Edamin^®^ K were identified to be crucial media components for an efficient production of erinacine C. The highest concentrations of erinacine C were obtained in the optimized culture medium on the 9^th^ culture day (approximately 260 mg L^−1^). The production of erinacine P was strongly time dependent. The maximum concentration of erinacine P of 184 mg L^−1^ was observed on the third culture day. Afterwards, the concentrations of erinacine P decreased while the concentrations of erinacine C steadily increased. Comparable results were obtained by HPTLC with UV detection and HPLC with diode-array detection (DAD) analyses. Direct bioautography allowed for an additional analysis of the antimicrobial activity of the secondary metabolites.

**Conclusions:**

The C and N sources oatmeal and Edamin^®^ K induced the formation of erinacine C. Detailed product formation kinetics of the erinacines C and P have been reported for the first time. HPTLC combined with the *Aliivibrio fischeri* bioassay allowed for an instant detection of cyathane diterpenoids in crude extracts and for an evaluation of the antimicrobial activity of the secondary metabolites directly on the plate.

**Electronic supplementary material:**

The online version of this article (doi:10.1186/s40694-015-0018-y) contains supplementary material, which is available to authorized users.

## Background

Basidiomycetes are the highest developed fungi. Many of them, if not all, synthesize biologically active compounds with medicinal properties in their fruiting bodies and mycelia. *Hericium erinaceus* is an edible mushroom that belongs to the family *Hericiaceae* and has been used for the treatment of, e.g., digestive diseases in traditional Chinese medicine for more than 1000 years. Various compounds isolated from *H. erinaceus*, high molecular weight compounds as well as small molecules, have shown a variety of beneficial functions, such as anticancer, anti-inflammatory, and immunomodulatory properties [1–5]. Hericenones and erinacines are the major bioactive low molecular weight compounds formed by fruiting bodies and submerged cultures of *H. erinaceus*, respectively. Several of these compounds have been shown to significantly induce nerve growth factor (NGF) synthesis and to protect neuronal cells against endoplasmic reticulum (ER) stress- or oxidative stress-induced cell death. Therefore, the consumption of *H. erinaceus,* and of dietary supplements derived thereof, has been suggested for the prevention and treatment of dementia, and especially of Alzheimer’s disease [1, 6–12].

**Fig. 1:**
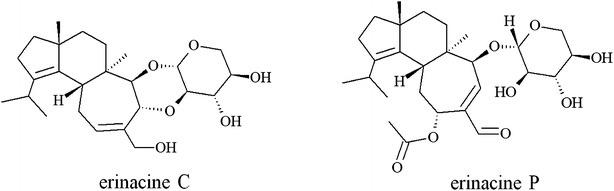
Structures of erinacine C and P

The erinacines A to I, P, and Q are diterpenoid compounds with a cyathane skeleton consisting of five-, six-, and seven-membered rings. Among the erinacines A to G, erinacine C (Fig. 1) showed the strongest induction of NGF synthesis [1]. Various strategies have been reported for the chemical synthesis of cyathane type diterpenoids, e.g., the total synthesis of allocyathin B_2_, erinacine A, and erinacine E [13–17]. However, all of the chemical syntheses are complex multistep processes and suffer from low yields. Watanabe and Nakada [15] described the total synthesis of erinacine E in 39 steps with a total yield of 0.9 %. Snider [17] reported the total synthesis of erinacine A in 19 steps with a yield of 1 %. Therefore, the biotechnological production of erinacine C was investigated in submerged cultures of *H. erinaceus* in the present study. The culture substrates were optimized and the production kinetics of erinacine C and of its supposed precursor erinacine P (Fig. 1) were recorded by HPLC–DAD analysis. Additionally, the combination of high-performance thin-layer chromatography (HPTLC) with a bioassay allowed for a fast evaluation of the antimicrobial activity of the erinacines.

## Results

### Optimization of erinacine C production

**Fig. 2:**
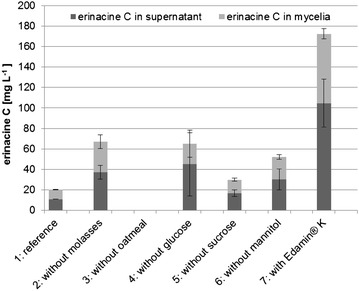
Effect of medium components on the production of erinacine C (means and standard deviations). The cultivation was stopped on day 27

The concentrations of erinacine C formed in the mycelia and supernatants of the cultures grown in media 1–7 were different (Fig. 2). The presence of oatmeal was crucial for the biosynthesis of erinacine C, as erinacine C was not detected in medium 3 (without oatmeal). Besides, the use of Edamin^®^ K increased the formation of erinacine C substantially. The concentration of erinacine C in medium 7, in which Edamin^®^ K was used instead of Edamin^®^ S, was about 8.5 times higher than in the reference medium 1.

**Fig. 3:**
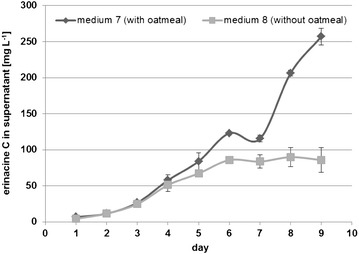
Production of erinacine C (means and standard deviations) in medium 7 (with oatmeal) *versus* medium 8 (without oatmeal)

Based on these results, the effect of oatmeal on erinacine C production was rechecked in medium 7 (with oatmeal) *versus* medium 8 (without oatmeal). Erinacine C was produced in medium 8 in spite of the missing oatmeal, but the production of erinacine C was higher in medium 7 (Fig. 3). Approximately 257 mg L^−1^ erinacine C was detected in the supernatants in medium 7 on the 9th culture day, compared to ~85 mg L^−1^ in the supernatants of medium 8. Therefore, medium 7 was chosen for the following experiments.

**Fig. 4:**
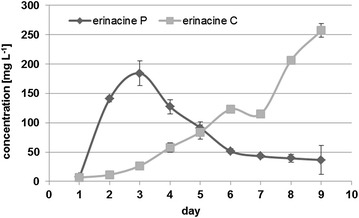
Production of erinacines C and P (means and standard deviations) in the culture supernatants of *H. erinaceus* (HPLC–DAD analysis at 210 nm, duplicate cultures)

The concentrations of erinacine P and C were both found to be time-dependent (Fig. 4). The concentrations of erinacine P increased during the first three culture days and decreased thereafter. With the decreasing concentrations of erinacine P, the concentrations of erinacine C increased steadily. A maximum concentration of erinacine P of ~184 mg L^−1^ was observed on the third culture day, while the maximum concentration of erinacine C was 257 mg L^−1^ on the 9th culture day.

### HPTLC-UV/vis/FLD-*Aliivibrio fischeri* analysis

HPLC has been mainly applied for the analysis of natural products from complex matrices. However, the use of HPTLC for natural product analysis provides a number of advantages. It allows, e.g., for a screening for bioactivity directly on the plate, for quantification, and by coupling to mass spectrometers also for compound confirmation or structure suggestions.

**Fig. 5:**
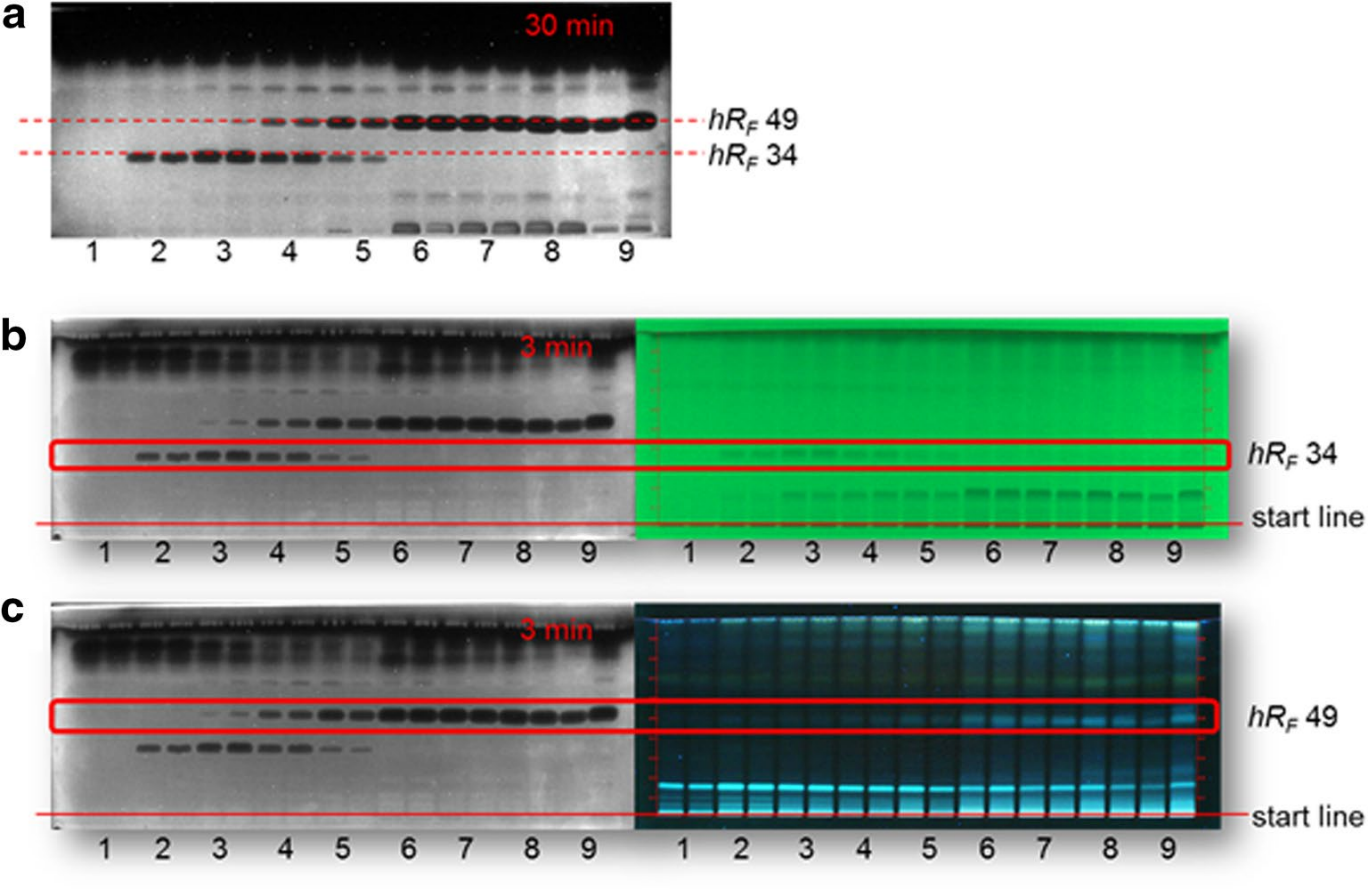
Direct bioautography (HPTLC-*Aliivibrio fischeri*) of secondary metabolites from submerged cultures of *H. erinaceus* from culture days 1–9 (applied in duplicate): **a** bioluminescent bioautogram documented as *grey scale* image after 30 min and after 3 min compared to detection at **b** UV 254 nm and **c** UV 366 nm

The compound zones at *hR*_F_ 34 (erinacine P) and *hR*_F_ 49 clearly showed antibacterial activity (Fig. 5). Erinacine P was also detectable by inspection of the chromatogram at UV 254 nm, while the substance at *hR*_F_ 49 was fluorescent at UV 366 nm. The results obtained by HPLC–DAD were clearly confirmed by HPTLC-UV/FLD analysis followed by bioautography. Also here, the concentrations of erinacine P increased during the first 3 cultivation days and decreased thereafter. It was evident that the unknown bioactive compound zones at *hR*_F_ 49 clearly increased, similarly to erinacine C as discussed before.

### HPTLC-mass spectrometry

**Fig. 6:**
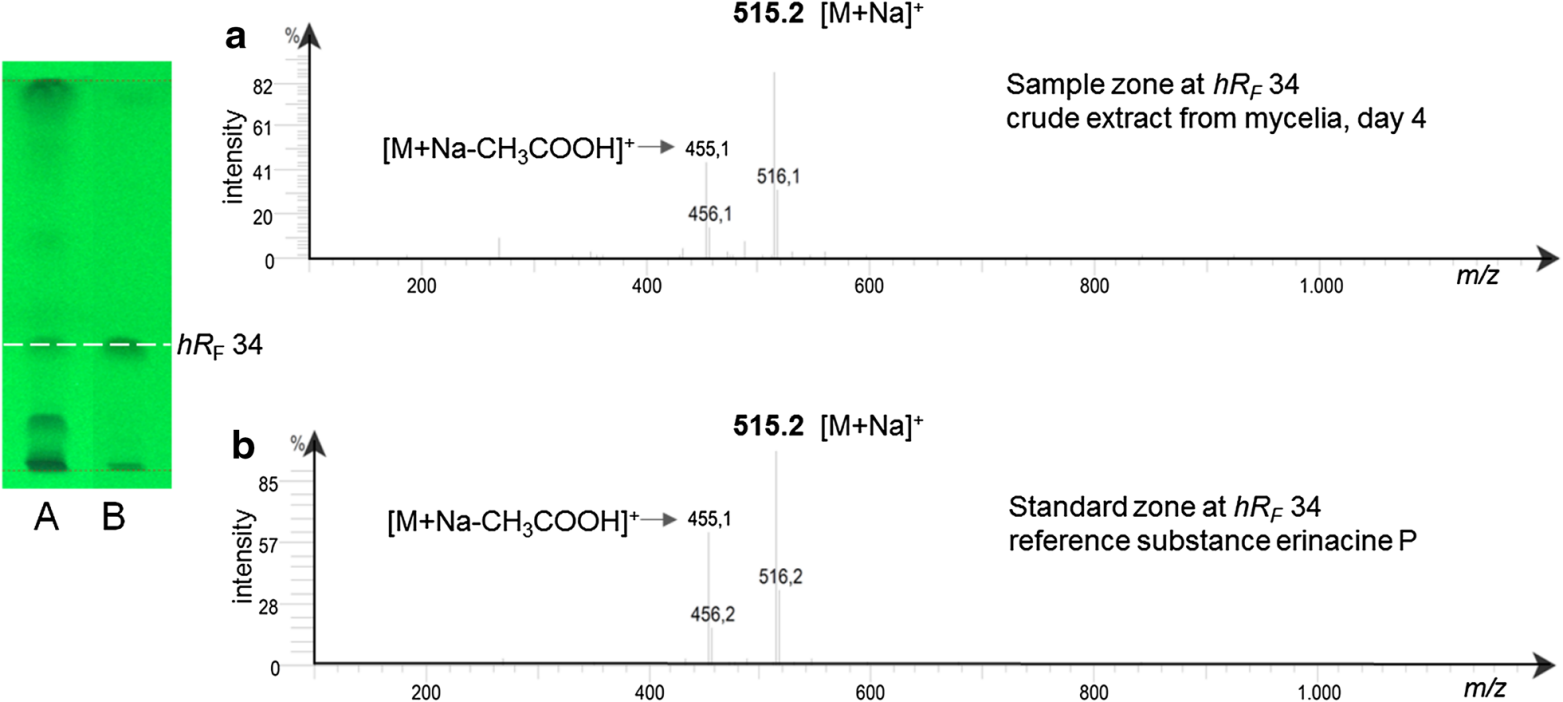
HPTLC-MS analysis. **a** Chromatogram and full scan MS spectra of the substance zone at *hR*_F_ 34 from crude mycelial extracts of culture day 4 in comparison to and **b** those of the reference erinacine P

Extracts of mycelia from submerged cultures of *H. erinaceus* on day 4 and the reference compound erinacine P were both applied on the same HPTLC plate. The MS spectra of the substance zone at *hR*_F_ 34 showed *m/z* values of 515.2 [M+Na]^+^ and 455.1 [M+Na–CH_3_COOH]^+^ in the ESI^+^ mode, which were identical with those of the reference compound erinacine P (Fig. 6).

## Discussion

The production of erinacine C strongly depended on the medium composition. Oatmeal and Edamin^®^ K supported the formation of erinacine C. Oatmeal as a complex substrate provided protein, fat, carbohydrates, dietary fiber, and mineral nutrients. It might act as a carbon source as well as a nitrogen source. Edamin^®^ K represents a high quality source of peptides and amino acids produced by enzymatic digestion of lactalbumin, which acted in the medium as a nitrogen source.

The comparison of medium 7 to medium 1 proved the essential role of Edamin^®^ K for the production of erinacine C. The maximum concentration of erinacine P of 184 mg L^−1^ was observed after 3 culture days (Fig. 4). Afterwards, the concentrations of erinacine P decreased while the concentrations of erinacine C steadily increased. These data are in good agreement with the results of Kenmoku et al. [12] who suggested erinacine P as a precursor of erinacine C. Erinacine P is supposed to be an early metabolite of the cyathane xylosides family as, e.g., the erinacines A and B were formed from erinacine P. The biosynthetic relationship between erinacine Q, erinacine P, and erinacine C was elucidated by a 1′-^13^C-d-glucose labeling experiment. Based on NMR data, 1′-^13^C-erinacine Q was converted into 1′-^13^C-erinacine C via 1′-^13^C-erinacine P [11]. The erinacines Q and P may be the common biosynthetic intermediates of cyathane xylosides such as the erinacines A, B, C and H in *H. erinaceus*, the erinacines E, F and G in *H. romosum*, and the striatals and striatins in *Cyathus* spp. However, detailed product formation kinetics of erinacine P and erinacine C have been reported in the present study for the first time. The product formation kinetics suggested an optimum harvest time of 3 days for erinacine P, and of 9 days for erinacine C. The product concentrations of >250 mg L^−1^ for erinacine C could form a basis for a profitable industrial fermentation process.

The production of erinacine C could be further optimized by, e.g., variation of the concentration of Edamin^®^ K, regulation of the culture pH, adjustment of an ideal C/N ratio or addition of certain mineral elements. Krzycykowski et al. [18] reported on the effects of the carbon and nitrogen sources, mineral elements, and of the initial pH of the culture medium on the growth of *H. erinaceus* and the production of erinacine A. A more acidic pH of the culture medium (i.e. pH 4.5) positively affected both mycelial growth and erinacine A production. Likewise, the addition of ZnSO_4_ and NaCl improved the synthesis of erinacine A. Considering the role of erinacine P as a precursor of cyathane xylosides, it may be necessary to optimize the yields of erinacine P to achieve higher product concentration of erinacine C.

HPTLC is commonly used in food analysis to detect, e.g., dyes, sugars, antioxidants, or artificial sweeteners. However, HPTLC is also broadly applied for the analysis of natural products. Recently, isolated sesquiterpenoids from *Cyperus rotundus* have been screened for their antioxidative potential [19]. The authors described a validated HPTLC method for the identification and quantification of solavetivone, aristolone and nootkatone, which showed no significant variation to the common HPLC method. The combination of HPTLC and bioassays (bioautography) or HPTLC and MS/NMR indicated that HPTLC might be considered as a fast and highly reliable method for compound identification or zone confirmation [20–24].

In the present study, HPTLC-UV/vis/FLD-bioassay was used in addition to HPLC–DAD for the analysis of secondary metabolites from submerged cultures of *H.* *erinaceus*. While erinacine C remained at the start line under the chromatographic conditions applied, the substance at *hR*_*F*_ 34 was confirmed to be erinacine P. This was substantiated by mass spectrometric data, UV/vis spectra, and comparison to the authentic reference compound. Comparable product formation kinetics for erinacine P were obtained by HPLC–DAD and HPTLC analyses (Additional file 1: Figures S1, S2). Different from HPLC–DAD, HPTLC allowed for visualization of antibacterial compounds directly on the plate. The antimicrobial activity of erinacine P has been described previously in [26]. Apart from erinacine P, a second compound with strong activity against *Aliivibrio fischeri* bacteria (*hR*_*F*_ 49) was detected in the supernatants of *H.* *erinaceus*. HPTLC-MS analyses did not allow for an unambiguous identification of this compound and of at least two further metabolites with antibacterial activity. Further HPTLC-high-resolution MS studies will be necessary to elucidate the structures of these metabolites.

## Conclusions

Oatmeal and Edamin^®^ K were found to be essential medium components for the production of erinacine C. The maximum concentration of erinacine P was observed after 3 culture days, while thereafter the concentration of erinacine C steadily increased. The proposed HPTLC-UV/vis/FLD-bioassay method allowed for an efficient detection and also quantification of erinacine P. Bioactive secondary metabolites from submerged cultures of *H. erinaceus* were observed instantly via direct bioautography (HPTLC-*Aliivibrio fischeri*). The direct link to antimicrobial compounds and confirmation by mass spectrometry was considered as streamlined tool for natural product analysis.

## Methods

### Fungal strain and reference compounds

*Hericium erinaceus* (FU70034, isolated from basidiocarp tissue) was obtained from InterMed Discovery (IMD) Natural Solutions, Dortmund, Germany. The fungus was maintained on a solid medium containing 20 g L^−1^ malt extract (Fluka, Neu-Ulm, Germany) and 15 g L^−1^ agar–agar (Roth, Karlsruhe, Germany). The identity of the strain was confirmed by comparison of its ITS nrDNA sequences with reference data in NCBI BLAST^®^ (for sequence data cf. Additional file 1: Figure S3). The strain FU70034 showed 95 % identity to *Hericium erinaceus* from different geographic origins (Additional file 1: Table S1 with references).

Reference substances of erinacine C and erinacine P were isolated from extracts of *H. erinaceus* cultures by preparative HPLC and identified by MS and NMR analyses as described previously [25].

### Medium composition and cultivation

Yeast malt medium consisted of d-(+)-glucose monohydrate 4.0 g L^−1^, malt extract 10.0 g L^−1^, and yeast extract 4.0 g L^−1^ (all Roth). The pH was adjusted to 6.3, and the medium was autoclaved at 121 °C for 20 min prior to use. Various main culture media were tested (data not shown), and the production of erinacine C was confirmed only in sugar molasses medium (medium 1). The optimization of erinacine C production was thus based on medium 1. One of the carbon sources was omitted in the media 2–7, respectively, and the nitrogen source Edamin^®^ S was changed to Edamin^®^ K in media 7 and 8 (Table 1). Molasses was obtained from Südzucker, Offstein, Germany and oatmeal was from Dr. Oetker, Düsseldorf, Germany. Sucrose and ammonium sulfate were purchased from Roth, and d-Mannitol, Edamin^®^ S, Edamin^®^ K, and calcium carbonate were purchased from Sigma, Taufkirchen, Germany. The media were autoclaved at 121 °C for 20 min prior to use.

**Table 1:** Main culture media 1–8 for optimization of erinacine C production

Medium concentration (g L^−1^)	**1**	**2**	**3**	**4**	**5**	**6**	**7**	**8**
Molasses	5	–	5	5	5	5	5	5
Oatmeal	5	5	–	5	5	5	5	–
d-(+)-glucose monohydrate	1.5	1.5	1.5	–	1.5	1.5	1.5	1.5
Sucrose	4	4	4	4	–	4	4	4
d-mannitol	4	4	4	4	4	–	4	4
Edamin^®^ S or K	0.5 S	0.5 S	0.5 S	0.5 S	0.5 S	0.5 S	0.5 K	0.5 K
Ammonium sulfate	0.5	0.5	0.5	0.5	0.5	0.5	0.5	0.5
Calcium carbonate	1.5	1.5	1.5	1.5	1.5	1.5	1.5	1.5

Pre-cultures of *H. erinaceus* were grown submerged in 250 mL Erlenmeyer flasks containing 100 mL yeast malt medium at 24 °C and 150 rpm for 7 days. Afterwards, 40 mL homogenized mycelia were inoculated into 400 mL main culture medium in 1000 mL Erlenmeyer flasks and incubated at 24 °C and 150 rpm for further 9 days. All samples for HPTLC analysis were obtained from cultures in medium 7.

### Sample preparation

Twenty mL culture supernatant were extracted with 20 mL ethyl acetate (≥99.5 %, Ph. Eur., Roth). The ethyl acetate phase was dried over sodium sulfate (water free, >99 %, Roth) and 15 mL of the extract was evaporated to dryness. The residue was dissolved in 1 mL acetonitrile (Chromasolv, gradient grade, Sigma) for HPLC and HPTLC analyses.

Additionally, the concentrations of erinacine C in the mycelia were analyzed by HPLC–DAD. Therefore, the mycelia were separated from the culture supernatants by centrifugation (2880×*g*, 4 °C, and 10 min) and washed 3 times with water. Afterwards, the mycelia were extracted with 20 mL ethyl acetate twice. The combined ethyl acetate phases were dried over sodium sulfate, and 30 mL of the extract was evaporated to dryness. The residue was dissolved in 1 mL acetonitrile for analysis.

Mycelia from culture day 4 were used for HPTLC-MS analyses. The mycelia were prepared as described before, except that 20 mL of the extract was evaporated to dryness. All experiments were performed in duplicate.

### HPLC–DAD analysis

The HPLC system consisted of pump L-7100, autosampler L-7200, interface D-7000, and diode array detector L-7455 (Merck Hitachi, Darmstadt, Germany). A reversed phase Nucleosil^®^ 100-5 C_18_, CC 125/3 mm with a respective guard column (CC 8/3 mm) was used (Macherey–Nagel, Düren, Germany). A mixture of acetonitrile (A) (HPLC gradient grade, Sigma) and bidistilled water (B) served as eluent. Gradient: 30 % A (0 min)—50 % A (15 min)—50 % A (16 min)—100 % A (23 min)—100 % A (38 min)—30 % A (43 min)—30 % A (47 min). The flow rate was 0.6 mL min^−1^.

### HPTLC-UV/vis/FLD analysis

HPTLC plates silica gel 60 F_254_, 20 cm × 10 cm, were obtained from Merck, Darmstadt, Germany. Prior to use, the plates were pre-washed with methanol—water (4:1, v/v) and dried (110 °C, 15 min). The sample extracts were applied as 8 mm bands onto the HPTLC plate using the Automatic TLC Sampler ATS 4 (CAMAG, Muttenz, Switzerland). The track distance was 10 mm. In the Twin Trough Chamber 20 × 10 cm (CAMAG), the plate was developed with 9 mL *n*-hexane–ethyl acetate–methanol (6:11:1, v/v/v). For an acidic plate conditioning, 1 mL acetic acid was filled in the opposite chamber trough. After development, the plate was dried at room temperature and documented at UV 254 nm, UV 366 nm, and white light illumination using the TLC Visualizer (CAMAG). The recording of spectra (200–700 nm) and densitometric absorbance measurement at 210 nm were performed by TLC Scanner 4 (CAMAG). All data were processed and evaluated by winCATS version 1.4.7.2018 (CAMAG).

### Bioautography

For bioautography, the HPTLC plate was automatically immersed into the bioluminescent *Aliivibrio fischeri* bacteria suspension prepared according to DIN EN ISO 11348-1, Sect. “Methods”. The immersion speed was 4 cm/s and the immersion time 1 s using the Chromatogram Immersion Device (CAMAG). The bioautogram was documented as greyscale image with an image accumulation time of 50 s using the BioLuminizer (CAMAG). Changes were monitored by capturing an image every 3 min over a 30-min period. *Aliivibrio fischeri* bioactive zones were instantly visible as darkened or enlightened zones on the luminescent background.

### HPTLC-mass spectrometry

On the chromatogram, the target zone erinacine P (*hR*_F_ 34) was marked at UV 254 nm with a soft pencil. The TLC-MS Interface equipped with the oval elution head of 4 mm × 2 mm (CAMAG) was coupled to the electrospray interface of a single quadrupole mass spectrometer (CMS, Advion, Ithaca, NY, USA). The target zone was eluted with methanol at a flow rate of 0.1 mL min^−1^. The total ion current (TIC) full scan mass spectra (*m/z* 100–1000) were recorded in the positive ionization mode. A plate/system background spectrum was recorded at a comparable migration distance and subtracted from the analyte spectrum. The elution head was flushed with eluent and dried after each measurement.

## Availability of supporting data

The data sets supporting the results of this article are included within the article and its additional file.

## Additional file

**Additional file 1.** Comparison of peak areas und UV-VIS spectra obtained for erinacine P by HPLC-DAD and by HPTLC-UV analyses.
