# Supplementary material for: Production of cyathane type secondary metabolites by submerged cultures of Hericium erinaceus and evaluation of their antibacterial activity by direct bioautography
Source: Fungal Biol Biotechnol. 2015 Dec 22;2:8. doi: 10.1186/s40694-015-0018-y (PMC5611583; doi:10.1186/s40694-015-0018-y)
Supplement: Supplementary file 1 — Additional file 1. Comparison of peak areas und UV-VIS spectra obtained for erinacine P by HPLC-DAD and by HPTLC-UV analyses. [file 40694_2015_18_MOESM1_ESM.docx]

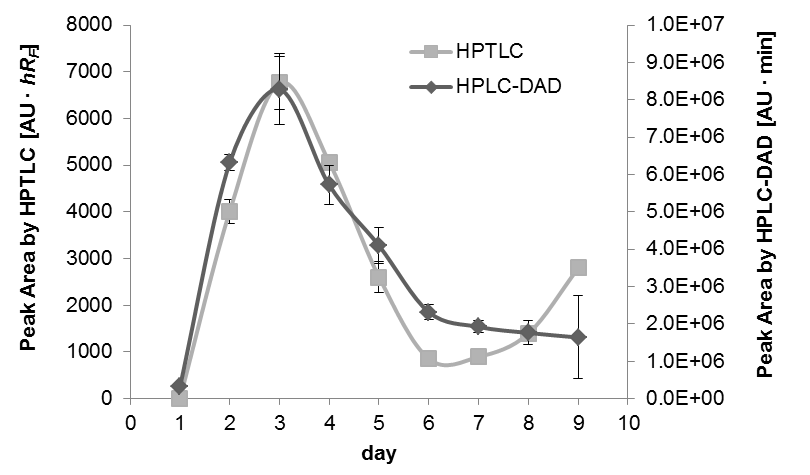


Supplementary Figure S1 Comparison of peak areas obtained for erinacine P (*hR*_F_ 34) by HPLC-DAD and by HPTLC-UV analyses.


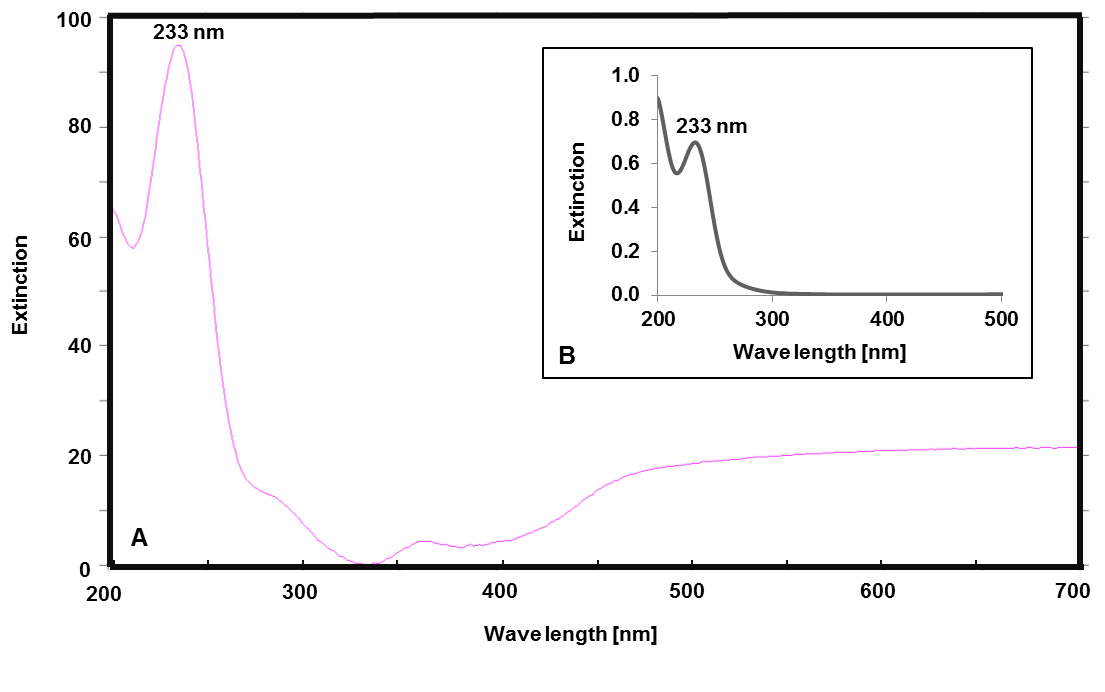


Supplementary Figure S2 UV/Vis spectra of (A) the bioactive substance zone at *hR*_F_ 34 assigned to be erinacine P, measured by HPTLC and (B) reference compound erinacine P, recorded by HPLC-DAD.

tcattaatgatttgaaaggagttgttgctggcctgaaacccaggcatgtgcatgctccaatctcatccatcttacacctgtgcacccttgcgtgggtccgtcggctttgcggtcgatgggcttgcgtttttcataaactcttacgcacgcaacgagaatgtcataatgctataaacgcatcttatacaactttcacaacggatctcttgctctcgcatcgatgaagaacgcagcgaaatgcgataagtaatgtgaattgcagaattcagtgaatcatcgaatctttgaacgcaccttgcgccccttgtattccgaggggcacgcctgtcgagtgtcgtgaaatctcaactcaatcctcttgtatgagagggttgggcttggacttggaggctcgccgtgctctcggaagtcggtctcttgaatcgcatcgat

Supplementary Figure S3 *Hericium erinaceus* strain FU70034, partial sequence, internal transcribed spacer 1, 5.8S ribosomal RNA gene.

Supplementary Table S1 Blast hits of FU70034, partial sequence, internal transcribed spacer 1, 5.8S ribosomal RNA gene and homologies to different strains of *H. erinaceus* [1-3].

| GenBank accession number | organism | geographic origin | identity | E-value |
| --- | --- | --- | --- | --- |
| AY534600 | *H. erinaceum** (KUMC 1022) | China | 95% | 0,0 |
| AY534601 | *H. erinaceum** (KUMC 1023) | Korea | 95% | 0,0 |
| AY534597 | *H. erinaceum** (KUMC Y-2) | Japan | 95% | 0,0 |
| AY534585 | *H. erinaceum** (NFCF F01) | Malaysia | 95% | 0,0 |
| AY534583 | *H. erinaceus* (CBS 485.95) | USA | 94% | 0,0 |
| EU784264 | *H. erinaceus* (K(M)61209) | UK | 95% | 0,0 |
| EU784265 | *H. erinaceus* (K(M)62494) | UK | 94% | 0,0 |
| AF397469 | *H. erinaceum** (HT_DB_) | China | 95% | 0,0 |

** H. erinaceum* is a synonym for *H. erinaceus*

References:

1. Park, H. G., H. G. Ko, S. H. Kim, W. M. Park: Molecular identification of asian isolates of medicinal mushroom *Hericium erinaceum* by phylogenetic analysis of the nuclear ITS rDNA. J Microbiol Biotechn. 2004;14:816–21.

2. Lu, L., J. Li, Y. Cang: PCR-based sensitive detection of medicinal fungi *Hericium* species from ribosomal internal transcribed spacer (ITS) sequences. Biol Pharm Bull. 2002; 25:975–80.

3. Brock, PM., H. Doring, M. I. Bidartondo: How to know unknown fungi: the role of a herbarium. New Phytol. 2009;181:719–24.
